# Measures of CNS-Autonomic Interaction and Responsiveness in Disorder of Consciousness

**DOI:** 10.3389/fnins.2019.00530

**Published:** 2019-06-21

**Authors:** Francesco Riganello, Stephen Karl Larroque, Carol Di Perri, Valeria Prada, Walter G. Sannita, Steven Laureys

**Affiliations:** ^1^Coma Science Group, GIGA-Consciousness, GIGA Institute, University Hospital of Liège, Liège, Belgium; ^2^S. Anna Institute, Research in Advanced Neurorehabilitation, Crotone, Italy; ^3^Centre for Clinical Brain Sciences, The University of Edinburgh, Edinburgh, United Kingdom; ^4^Department of Neuroscience, Rehabilitation, Ophthalmology, Genetics, and Maternal/Child Sciences, Polyclinic Hospital San Martino IRCCS, University of Genoa, Genoa, Italy

**Keywords:** central autonomic network, autonomic nervous system, disorders of consciousness, unresponsive wakefulness syndrome, heart rate variability

## Abstract

Neuroimaging studies have demonstrated functional interactions between autonomic (ANS) and brain (CNS) structures involved in higher brain functions, including attention and conscious processes. These interactions have been described by the Central Autonomic Network (CAN), a concept model based on the brain-heart two-way integrated interaction. Heart rate variability (HRV) measures proved reliable as non-invasive descriptors of the ANS-CNS function setup and are thought to reflect higher brain functions. Autonomic function, ANS-mediated responsiveness and the ANS-CNS interaction qualify as possible independent indicators for clinical functional assessment and prognosis in Disorders of Consciousness (DoC). HRV has proved helpful to investigate residual responsiveness in DoC and predict clinical recovery. Variability due to internal (e.g., homeostatic and circadian processes) and environmental factors remains a key independent variable and systematic research with this regard is warranted. The interest in bidirectional ANS-CNS interactions in a variety of physiopathological conditions is growing, however, these interactions have not been extensively investigated in DoC. In this brief review we illustrate the potentiality of brain-heart investigation by means of HRV analysis in assessing patients with DoC. The authors’ opinion is that this easy, inexpensive and non-invasive approach may provide useful information in the clinical assessment of this challenging patient population.

## Introduction

Clinical evidence and neuroimaging research have documented retained modular brain activation and responsiveness in patients with Disorder of Consciousness (DoC) following brain injury even in the absence of integrated large-network processes known to sustain consciousness (Laureys et al., 2002; Bekinschtein et al., 2004, 2011; Owen et al., 2006; Monti, 2012; Naro et al., 2015; Box 1). In this respect, residual responsiveness in DoC appears to be mediated by varying network interactions (Riganello et al., 2013, 2015c; Crone et al., 2017; Duclos et al., 2017). Activation restricted to lower-level primary sensory cortices without involvement of higher-order associative cortices has been described in vegetative state/unresponsive wakefulness syndrome (VS/UWS) (Soddu et al., 2015; Marino et al., 2017). Partially preserved activation in higher-order associative cortices has been demonstrated in Minimally Conscious State (MCS) (Di Perri et al., 2013, 2016; Demertzi et al., 2015), whereas restoration of thalamocortical connectivity has shown to relate to consciousness recovery (Laureys et al., 2000; Monti et al., 2014). A large amount of research by means of neuroimaging techniques has revealed that several aspects of relatively high-level functions, including sensory and linguistic processing and learning dynamics, can survive and remain operative in DoC (Aubinet C- HBM 2018, Laureys et al., 2007; Boly et al., 2008; Majerus et al., 2009; Bruno et al., 2010).

**Box 1. HSS model predictions.**

Brain injury can result in a vegetative state/unresponsive wakefulness syndrome (VS/UWS) characterized by arousal and spontaneous eye-opening in the absence of any sign of awareness, finalized action or communication. Levels of residual responsiveness define the Minimally Conscious State (MCS) (Giacino et al., 2004). A 2006 provocative report presented the case of a VS/UWS subject able to engage in mental tasks as indicated by her fMRI patterns of brain activations (Owen et al., 2006). Levels of responsiveness involving higher brain functions have been observed in subjects otherwise classified as VS/UWS according to clinical criteria (Laureys et al., 2007; Boly et al., 2008; Bruno et al., 2010; Owen, 2014; Pistoia et al., 2016; Riganello et al., 2018b). These observations were mostly based on regional brain activation in response to stimulus conditions in controlled setups; stimulus-related functional changes in the autonomic nervous system (ANS) function have also been described. Still highly debated, e.g., in subjects in a VS/UWS, these observations challenge the current definitions and our understanding of both responsiveness and consciousness, with an impact on the clinical decision-making process (Laureys et al., 2010; Riganello et al., 2016, 2018a, 2018b). The extent to which regional brain activations can be considered equivalent to, or compatible with behavioral responses in indicating (residual or covert) consciousness remains controversial and the current standards by which patients surviving severe brain injury should be regarded as being conscious or unconscious have been questioned (Celesia, 2013; Celesia and Sannita, 2013). In this respect, scientific research has introduced novel criteria of evaluation not yet fully integrated in the current nosography of disorders of consciousness (DoC), which is now undergoing a tacit, but not uncontroversial, revision (Monti and Sannita, 2016).

Neuroimaging studies have further shown functional interaction between autonomic nervous structures [i.e., the parasympathetic and sympathetic branch of the Autonomic Nervous System (ANS)] and the neuronal networks involved in higher brain functions, including attention and conscious processes (Napadow et al., 2008; Thayer et al., 2012; Ruiz Vargas et al., 2016; Valenza et al., 2017). Heart Rate Variability (HRV), that is the physiological phenomenon of variation in the time interval between consecutive heartbeats, is thought to reflect the complex interaction between brain and cardiovascular system (Thayer and Lane, 2009; Ernst, 2017). HRV entropy, a measure of the complexity of HRV, has shown to discriminate VS/UWS and MCS patients and was found to correlate with the ANS functional status (Riganello et al., 2018b).

In agreement with this line of observation, indices of ANS functions have proved reliable in detecting responsiveness and predicting recovery following neuro-rehabilitation in VS/UWS (Wijnen et al., 2006; Riganello et al., 2015a). There is growing evidence that ANS function can be monitored non-invasively and neuroimaging studies have provided evidence of the two-way interplay between heart and brain. As a result, interest in the bidirectional ANS-CNS interaction in a variety of physiopathological conditions is growing (de Morree et al., 2013; Riganello et al., 2014; Bassi and Bozzali, 2015; Chen et al., 2017; Doehner et al., 2018), however, the ANS-CNS interaction in DoC has so far not been extensively investigated.

## ANS-CNS Interaction in DOC

**FIGURE 1:**
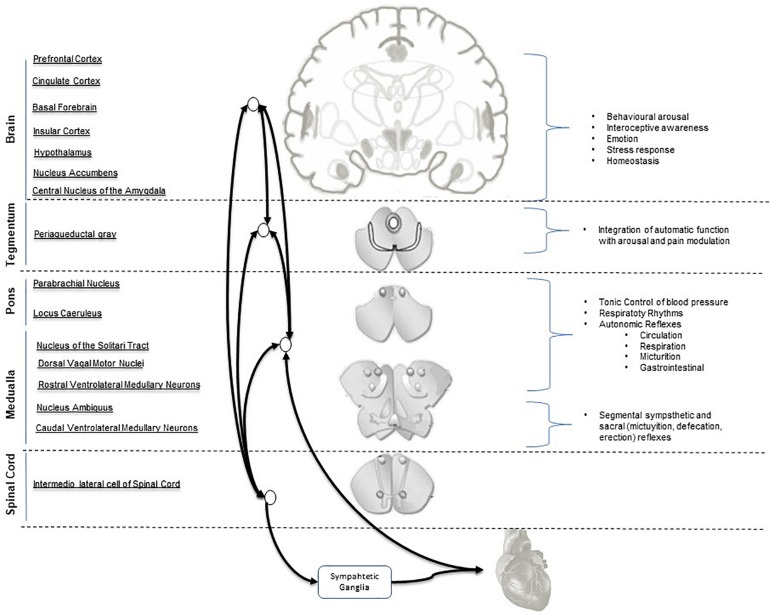
Central Autonomic Network (CAN) hierarchical organization and interconnections. Parasympathetic output is mediated mainly by the nucleus of the vagus and nucleus ambiguous. Sympathetic output is mainly mediated by the intermediolateral column cell.

A concept model Central Autonomic Network (CAN) (Benarroch, 2007b) has been proposed to describe the ANS-CNS two-way interaction and the continuous modulation of homeostatic processes and allostatic adaptation to internal or external requirements (Friedman, 2007; Thayer et al., 2012; Riganello, 2016). Its functional organization involves the forebrain (anterior cingulate, nucleus accumbens, insula, ventromedial prefrontal cortex, amygdala, and hypothalamus with bidirectional interactions between rostral and caudal systems), brainstem (periaqueductal gray, parabrachial nucleus, nucleus of the solitary tract, and the reticular formation of ventrolateral medulla). At spinal level it operates via neuronal projections of segmental reflexive ANS control (Figure 1). These structures receive converging visceral and nociceptive inputs (including those from thermo- and muscle receptors) and generate stimulus-specific patterns of autonomic response via projections to preganglionic sympathetic and parasympathetic neurons (Saper, 2002; Benarroch, 2007a). The forebrain and brainstem are involved in the modulation of autonomic output in response to pain and to emotional, behavioral, or “cognitive” stimuli (Hagemann et al., 2003; Berntson and Cacioppo, 2004; Thayer and Sternberg, 2006; Friedman, 2007; Thayer and Lane, 2009; Riganello et al., 2012a).

## Heart Rate Variability and Heart/Brain Interplay

HRV measures (i.e., variables analyzed in time domain, frequency domain and non-linear measurements) describe the ANS functional setup, and are thought to reflect higher brain functions - at least to some extent - and to qualify as independent indicators of CNS-ANS interaction (Napadow et al., 2008; Thayer and Lane, 2009; Thayer et al., 2012; Tonhajzerova et al., 2012) (Box 2 and Table 1). HRV measures reflect the activity of physiological factors modulating the heart rhythm and its adaptation to changing conditions (Carney et al., 2005; Garan, 2009; Shaffer et al., 2014). The vagus nerve is the major channel involved in the afferent neurological signals relayed from the heart and other visceral organs to the brain, including the baroreflex signals (de Lartigue, 2014). Brain morphological variants in the right striatal and limbic structures involved in the ANS functional organization were found to associate with differences in cardiac vagal function (Thayer and Lane, 2000; Napadow et al., 2008; Critchley, 2009; Lane et al., 2009) and to significantly contribute in the information flow in all frequency bands during sleep (Faes et al., 2014). In the absence of cardiac disorders, stimulus- or condition-related HRV changes are in the range of physiological variability and require processing in the time and frequency domains or by geometrical or non-linear methods to be identified (Task Force of the European Society of Cardiology and the North American Society of Pacing and Electrophysiology, 1996; Rajendra Acharya et al., 2006). HRV methodologies benefit from being easy to record, inexpensive and non-invasive as compared to neuroimaging techniques. HRV measures are obtained from a signal (the heart tachogram) with excellent signal-to-noise ratio; procedures for sophisticated data analyses are usable without substantial loss in information (Nait-Ali, 2009). HRV is easier to apply than alternative techniques for ANS investigation (cardiac norepinephrine spillover, microneurographic techniques, or direct recording from skeletal muscle) (Berne et al., 1992; Esler, 1993; Wallin and Charkoudian, 2007). It is particularly applicable in studies on large subject samples or under experimental conditions where accurate laboratory procedures are not possible, such as in case of limited/null collaboration (e.g., in intensive/semi-intensive care units or in DoC) (Mowery et al., 2008; Norris et al., 2008a, 2008b; Ryan et al., 2011).

**Box 2. HSS model predictions.**

HRV – Heart rate variability is defined as the R-R interval fluctuation of normal sinus beats around its average value. HRV is analyzed in time domain, frequency domain and by non-linear methods both in short (usually 5 min) and long-time recordings (Task Force of the European Society of Cardiology and the North American Society of Pacing and Electrophysiology, 1996). ANS functional contributions can be differentiated by analyses in the frequency domain, usually in three frequency band: High Frequency (HF) (0.15–0.5 Hz), Low Frequency (LF) (0.04–0.15 HZ), and (VLF) Very Low Frequency band (0.0033–0.04). The power, relative power and peak of each frequency band, the normalized value of HF (nuHF) and LF (nuLF) and the ratio between HF and LF (LF/HF) are the parameters usually considered. HF reflects parasympathetic activity as the HR variations related to the respiratory cycle. LF (0.04–0.15 Hz) mainly reflects baroreceptor activity during resting conditions and is known also as “baroreceptor range” (Malliani, 1995); it reflects contributions from both the parasympathetic and sympathetic systems and blood pressure regulation via baroreceptors and the baroreceptor activity in resting conditions (Malliani, 1995; Task Force of the European Society of Cardiology and the North American Society of Pacing and Electrophysiology, 1996; Berntson et al., 2007; Lehrer, 2007). The parasympathetic system affects heart rhythms down to 0.05 Hz, while the sympathetic system does not seem to generate rhythms above ∼0.1 Hz. In humans, the delay in the feedback loops of baroreflex system have distinctive high-amplitude peak in the HRV power spectrum around 0.1 Hz (Vaschillo et al., 2011; Lehrer and Eddie, 2013) due to the feedback loops between heart and brain (deBoer et al., 1987; Baselli et al., 1994). There is some evidence for an association between VLF band (0.0033–0.04 Hz) and all-cause mortality (Shaffer et al., 2014), however, the physiological mechanisms responsible for activity within this band are not clear although probably related to thermoregulation, the renin-angiotensin system, and other hormonal factors (Akselrod et al., 1981; Cerutti, 1995; Claydon and Krassioukov, 2008). The LF/HF ratio has controversial interpretations, with the LF power possibly generated by the sympathetic, the HF power by the parasympathetic system and their ratio indicating both parasympathetic or sympathetic dominance (Pagani et al., 1984; Montano et al., 1994). HRV descriptors are also derivable by non-linear methods. Development in the non-linear theories provides new instruments to analyse the entropy domain [such as the simple or approximate entropy (ApEn)], better describe the complexity, irregularity or randomness of HRV and its changes, the non-linear temporal relationships with other metrics such as functional connectivity, and extract information about the complexity of the two way brain-heart interaction (Norris et al., 2006; Ryan et al., 2011; Shaffer et al., 2014; Riganello et al., 2018b).

**TABLE 1:** Most common measures used in HRV analysis.

	**Parameter**	**Unit**	**Description**	
**Time domain**	SDNN/SDRR	ms	Standard deviation of NN/RR intervals	In the time domain both sympathetic and parasympathetic nervous systems contribute to SDNN. Differently from “RR,” ”NN” means that abnormal beats, like ectopic beats, have been removed. RMSSD is used to estimate the vagally mediated changes reflected in HRV. It is strongly correlated with pNN50
	SDANN		Standard deviation of the average normal-to-normal (NN) intervals for each of the 5 min segments during a 24 h recording	
	SDNN index		Mean of the standard deviations of all the NN intervals for each 5 min segment of a 24-h HRV recording	
	pNN50	%	Percentage of adjacent NN intervals that differ from each other by more than 50 ms (pNN50) (Task Force of the European Society of Cardiology and the North American Society of Pacing and Electrophysiology, 1996)	
	RMSSD	ms	Root mean square of successive differences between normal heartbeats	
**Frequency Domain**	VLF,LF, and HF power HF power	ms^2^	Absolute power of total or of the single band of frequency calculated by FFT or Auto Regressive model	HF, LF and VLF bands of frequency are associated with several aspects of the ANS. The HF band reflects parasympathetic activity and corresponds to the HR variations related to the respiratory cycle. The LF band reflects contributions from both the parasympathetic and sympathetic systems. It mainly reflects baroreceptor activity during resting conditions. The sympathetic system is below 0.1 Hz. The VLF band is associated to thermoregulation, the renin-angiotensin system, and other hormonal factors, but also to the intrinsic heart activity. The normalized values (nuLF and nuHF) express the quantities on a more easily understood proportion (0–1) or percentage (0–100%) scale basis. The LF/HF ratio is generally used to represents the ratio of sympathetic to parasympathetic nerve activity, also if the LF is contaminated by the vagal system. nuLF, nuHF, as well as LF/HF ratio should be considered equivalent carriers of information with regard to sympathovagal balance (Burr, 2007)
	nuLF	nu	Relative power of the low-frequency band in normal unit	
	nuHF		Relative power of the high-frequency band in normal unit	
	LF/HF		Ratio LF-to-HF power	
	Peak VLF, LF, and HF	Hz	Peak of frequency of VLF, LF, or HF band	
**Non-linear methods**	Approximate entropy		Measures the regularity and complexity of a time series	The entropy reflects of the amount of irregularity in the R-to-R interval. Lower or higher values are index of higher or lower complex activity of the ANS. Further higher values were associated to a higher brain-heart two way interaction. The SampEn was introduced to counteract some shortcomings of the ApEn. The SampEn does not count a self-match of vectors, eliminating the bias toward regularity, and has been suggested to be independent of data length (Yentes et al., 2013)
	Sample entropy		Measures the regularity and complexity of a time series. Sample entropy can be calculated from a much shorter time series of fewer than 200 values	
	Multiscale entropy		Quantify the degree of irregularity over a range of time scales. The time series are constructed by averaging the IBI/tachogram’s data points within non-overlapping windows of increasing length	

## HRV and DOC

HRV is a possible index of consciousness attention (Babo-Rebelo et al., 2016; Cobos et al., 2019) and emotional states (Shi et al., 2017) in healthy subjects. The interaction between consciousness, attention and HRV has been documented in patients with DoC. VS/UWS and MCS subjects were more likely to respond to standard visual and auditory stimuli when the HRV normalized unit of LF (nuLF) ranged between 10–70 and LF peaked around 0.1 Hz (Riganello et al., 2013), thus suggesting a functional relationship between responsiveness and the sympatho-vagal balance; a correlation between HRV parameters and improvement of consciousness has been documented, and higher value of nuLF associated to a better outcome in VS/UWS patients (Riganello et al., 2015a; Wijnen et al., 2006).

In frequency domain, HRV total power (TP) as well as LF and HF power were found significantly decreased in patients with Glasgow Coma Outcome Extended (GOSE) (Weir et al., 2012) score <5 (Hendén et al., 2014).

A correlation between HRV entropy (index of the brain-heart interaction complexity) and brain activation has been also described. Approximate Entropy (ApEn) values were lower in VS/UWS patients than in healthy control whereas no differences were found for all linear parameters [Root Mean Square of Successive Differences between normal heartbeats (RMSSD), Standard Deviation of RR peak (SDRR)] (Sarà et al., 2008). We have recently found lower Multiscale Entropy (MSE) values in VS/UWS than in MCS, which correlated with the Coma Recovery Scale (CSR-R) total score. A functional connectivity pattern involving the CAN system has been documented, thus proposing HRV entropy as an indirect tool to measure and monitor connectivity changes in this neural system (Riganello et al., 2018b). In a Evoked Response Potential (ERP) study based on nociceptive repeated laser stimulation MCS showed partially preserved cortical activations, higher ERP γ-power magnitude and Standard Deviation of Normal-to-Normal intervals (SDNN) compared to VS/UWS patients (Calabrò et al., 2017). Other studies on nociceptive stimulation documented a correlation between the level of consciousness and HRV-entropy (Riganello et al., 2018a; Tobaldini et al., 2018), with lower values of entropy detected in VS/UWS than in MCS patients or in healthy subjects, and correlated to the CRS-R total scores (Riganello et al., 2018a). The above results indicate a decrease of complexity in the modulation of the response to noxious stimuli in VS/UWS subjects and a less complex ANS modulation in the two way brain-heart interaction compared to MCS.

Similar results have been observed in response to complex (musical) stimuli. Music interventions were associated with favorable behavioral and physiological responses in several studies, however, methodological quality and outcomes were heterogeneous (Grimm and Kreutz, 2018). After 14-day of music stimulation SDNN and RMSSD of VS/UWS patients increased, indicating the activity of the cardiovascular system was enhanced (Lee et al., 2011). Contrasting observations were found in the direction of the RMSSD values in a work on MCS and VS/UWS patient, who were presented live preferred music and live improvised music (O’Kelly and Magee, 2013). A significant decrease in entropy was observed in VS/UWS subjects listening to four musical pieces of different structural complexity, whereas no differences between the same selected musical pieces were observed in healthy controls under comparable experimental conditions (Riganello et al., 2015b). The quality (positive or negative) of the emotional responses was correlated to extreme (low or high) nuLF values (Riganello et al., 2010). Higher values in both time and frequency domain were observed during affective than during non-affective auditory stimulation in VS/UWS patients suggesting the possibility to discriminate between different stimuli (Machado et al., 2007; Gutiérrez et al., 2010).

The clinical and scientific evidence suggests a diagnostic and prognostic relevance of HRV parameters in DoC of different aetiologies (e.g., traumatic brain injury (TBI), haemorrhagic, and anoxic) (Keren et al., 2005; King et al., 2009; Ryan et al., 2011; Almeida et al., 2017). Decreased values in the different domains of HRV analysis has been associated with worsened heath condition. HRV parameters extracted in the time domain (SDNN, SDNN index, and RMSSD) were also found decreased after TBI in the absence of major DoC (Rapenne et al., 2001; DeGiorgio et al., 2010; Kim et al., 2017) and associated to clinical worsening and to mortality in the acute phase (Morris et al., 2006; Norris et al., 2006; Mowery et al., 2008; King et al., 2009). In children, suppression of LF and HF bands of the power spectrum were associated with brain death and poor outcome (Goldstein et al., 1993, 1998) and decreases in LF/HF was correlated with increases in intracranial pressure and mortality (Biswas et al., 2000). In TBI adults, decreased LF, HF, LF/HF, and TP were associated with brain death, increased mortality, increased intracranial pressure, and poor outcome (Winchell and Hoyt, 1997; Rapenne et al., 2001; Papaioannou et al., 2006). Reduced HRV complexity has proved to be an independent predictor of mortality (Batchinsky et al., 2007). Decreased ApEn values have been associated to increased mortality in acute TBI (Papaioannou et al., 2008; Gao et al., 2016) and the MSE was found to identify trauma patients at risk of in-hospital death, and predicts mortality independent of probability of survival based on location and mechanism of injury (Norris et al., 2008a, 2008b).

## Comment and Perspectives

The CAN model of ANS-CNS functional interaction is helpful to describe the phenomena underlying residual responsiveness in DoC within the framework of homeostatic and allostatic organization, at least in part and to a degree of pathophysiological approximation (Friedman, 2007; Shen et al., 2016; Thome et al., 2017). The suitability of HRV analysis in detecting residual (covert) brain function in DoC has been documented (Wijnen et al., 2006; Gutiérrez et al., 2010; Riganello, 2016; Garbarino and Sannita, 2015). Autonomic function, ANS-mediated responsiveness and the ANS-CNS interaction qualify as possible independent indicators for clinical functional assessment, diagnosis and prognosis in DoC (King et al., 2009; Ryan et al., 2011; Sannita, 2015; Riganello, 2016). In a reversed perspective, research on the residual modular functions in DoC can provide unique information about brain mechanisms/functions and ANS-CNS interplay that can be investigated in these patients under experimental conditions that are rigorously controlled (Monti, 2012; Riganello et al., 2012b; Sannita, 2014; Shen et al., 2016; Chennu et al., 2017; Kiryachkov et al., 2017).

Brain function is modulated by complex neural networks and non-neuronal factors which interact with each other, individually or collectively account for inter/intra-individual variability, and reflect/depend on the circadian rhythms and the wakefulness/sleep alternation (Bullock, 1970; Sannita, 2006; Garbarino et al., 2014, 2019; Soddu and Bassetti, 2017). The HRV concomitants of the major shift toward sympathetic activation associated to peak cortisol levels at the morning sleep-to-wake transition are an example in this regard (Bilan et al., 2005; Boudreau et al., 2011, 2012). HRV proved reliable in investigating the ANS-CNS functional interaction underlying residual responsiveness in VS/UWS or MCS subjects (Wijnen et al., 2006; Gutiérrez et al., 2010; Candelieri et al., 2011; Sannita, 2015; Riganello, 2016). CNS and ANS setups, however, change over time spontaneously or due to homeostatic or allostatic requirements with different timing and latencies. HRV measures at rest and in response to stimulus conditions have higher time resolution and reflect rapid changes better than clinical or neuroimaging markers of damage, with greater variability during the day (Bekinschtein et al., 2009; Candelieri et al., 2011; Riganello et al., 2013, 2015c; Abbate et al., 2014; Sannita, 2015; Blume et al., 2017). Time appears to be a source of variability adding to the variety of environmental factors (light and noise in hospital settings, timing of medication or non-pharmacologic interventions, co-morbidities, etc.) also needing consideration, both as co-determinants of the circadian rhythms (Soddu and Bassetti, 2017) and in view of the ANS major role in internal environment constancy and adaptation that are fundamental to homeostasis. Systematic investigation is still lacking and appears advisable.

## Author Contributions

FR, SKL, CDP, VP, WS, and SL have equally collaborate to this work with substantial, direct and intellectual contribute, and approved it for publication.

## Conflict of Interest Statement

The authors declare that the research was conducted in the absence of any commercial or financial relationships that could be construed as a potential conflict of interest.

## References

[B1] Abbate C. Trimarchi P. D. Basile I. Mazzucchi A. Devalle G. (2014). Sensory stimulation for patients with disorders of consciousness: from stimulation to rehabilitation. *Front. Hum. Neurosci.* 8:616 10.3389/fnhum.2014.00616 PMC4127462 25157226

[B2] Akselrod S. Gordon D. Ubel F. A. Shannon D. C. Berger A. C. Cohen R. J. (1981). Power spectrum analysis of heart rate fluctuation: a quantitative probe of beat-to-beat cardiovascular control. *Science* 213 220–222. 10.1126/science.6166045 6166045

[B3] Almeida R. Dias C. Silva M. E. Rocha A. P. (2017). “ARFIMA-GARCH modeling of HRV: clinical application in acute brain injury,” in *Complexity and Nonlinearity in Cardiovascular Signals*, eds Barbieri R. Scilingo E. P. Valenza G. (Cham: Springer), 451–468. 10.1007/978-3-319-58709-7_17

[B4] Babo-Rebelo M. Richter C. G. Tallon-Baudry C. (2016). Neural responses to heartbeats in the default network encode the self in spontaneous thoughts. *J. Neurosci.* 36 7829–7840. 10.1523/JNEUROSCI.0262-16.2016 27466329 PMC4961773

[B5] Baselli G. Cerutti S. Badilini F. Biancardi L. Porta A. Pagani M. (1994). Model for the assessment of heart period and arterial pressure variability interactions and of respiration influences. *Med. Biol. Eng. Comput.* 32 143–152. 10.1007/bf02518911 8022210

[B6] Bassi A. Bozzali M. (2015). Potential interactions between the autonomic nervous system and higher level functions in neurological and neuropsychiatric conditions. *Front. Neurol.* 6:182. 10.3389/fneur.2015.00182 26388831 PMC4559639

[B7] Batchinsky A. I. Cancio L. C. Salinas J. Kuusela T. Cooke W. H. Wang J. J. (2007). Prehospital loss of R-to-R interval complexity is associated with mortality in trauma patients. *J. Trauma* 63 512–518. 10.1097/TA.0b013e318142d2f0 18073594

[B8] Bekinschtein T. Niklison J. Sigman L. Manes F. Leiguarda R. Armony J. (2004). Emotion processing in the minimally conscious state. *J. Neurol. Neurosurg. Psychiatry* 75 788–788. 10.1136/jnnp.2003.034876 15090585 PMC1763566

[B9] Bekinschtein T. A. Golombek D. A. Simonetta S. H. Coleman M. R. Manes F. F. (2009). Circadian rhythms in the vegetative state. *Brain Inj.* 23 915–919. 10.1080/02699050903283197 20100128

[B10] Bekinschtein T. A. Manes F. F. Villarreal M. Owen A. M. Della Maggiore V. (2011). Functional imaging reveals movement preparatory activity in the vegetative state. *Front. Hum. Neurosci.* 5:5. 10.3389/fnhum.2011.00005 21441977 PMC3031991

[B11] Benarroch E. E. (2007a). Enteric nervous system Functional organization and neurologic implications. *Neurology* 69 1953–1957. 10.1212/01.wnl.0000281999.56102.b5 17998487

[B12] Benarroch E. E. (2007b). The autonomic nervous system: basic anatomy and physiology. *Contin. Lifelong Learn. Neurol.* 13 13–32. 10.1212/01.CON.0000299964.20642.9a

[B13] Berne C. Fagius J. Pollare T. Hjemdahl P. (1992). The sympathetic response to euglycaemic hyperinsulinaemia. *Diabetologia* 35 873–879. 10.1007/BF00399935 1397783

[B14] Berntson G. G. Cacioppo J. T. (2004). “Heart rate variability: stress and psychiatric conditions,” in *Dynamic Electrocardiography*, eds Malik M. Camm A. J. (New York, NY: Blackwell/Futura), 57–64.

[B15] Berntson G. G. Cacioppo J. T. Grossman P. (2007). Whither vagal tone. *Biol. Psychol.* 74 295–300. 10.1016/j.biopsycho.2006.08.006 17046142

[B16] Bilan A. Witczak A. Palusiński R. Myśliński W. Hanzlik J. (2005). Circadian rhythm of spectral indices of heart rate variability in healthy subjects. *J. Electrocardiol.* 38 239–243. 10.1016/j.jelectrocard.2005.01.012 16003709

[B17] Biswas A. K. Scott W. A. Sommerauer J. F. Luckett P. M. (2000). Heart rate variability after acute traumatic brain injury in children. *Crit. Care Med.* 28 3907–3912. 10.1097/00003246-200012000-00030 11153634

[B18] Blume C. Lechinger J. Santhi N. del Giudice R Gnjezda M. T. Pichler G. (2017). Significance of circadian rhythms in severely brain-injured patients: A clue to consciousness? *Neurology* 88 1933–1941. 10.1212/WNL.0000000000003942 28424270 PMC5444311

[B19] Boly M. Faymonville M.-E. Schnakers C. Peigneux P. Lambermont B. Phillips C. (2008). Perception of pain in the minimally conscious state with PET activation: an observational study. *Lancet Neurol.* 7 1013–1020. 10.1016/S1474-4422(08)70219-9 18835749

[B20] Boudreau P. Dumont G. Kin N. M. Walker C.-D. Boivin D. B. (2011). Correlation of heart rate variability and circadian markers in humans. *Conf. Proc. IEEE Eng. Med. Biol. Soc.* 2011 681–682. 22254401 10.1109/IEMBS.2011.6090153

[B21] Boudreau P. Yeh W. H. Dumont G. A. Boivin D. B. (2012). A circadian rhythm in heart rate variability contributes to the increased cardiac sympathovagal response to awakening in the morning. *Chronobiol. Int.* 29 757–768. 10.3109/07420528.2012.674592 22734576

[B22] Bruno M.-A. Vanhaudenhuyse A. Schnakers C. Boly M. Gosseries O. Demertzi A. (2010). Visual fixation in the vegetative state: an observational case series PET study. *BMC Neurol.* 10:35. 10.1186/1471-2377-10-35 20504324 PMC2895583

[B23] Bullock T. H. (1970). The reliability of neurons. *J. Gen. Physiol.* 55 565–584. 10.1085/jgp.55.5.565 5462670 PMC2203020

[B24] Burr R. L. (2007). Interpretation of normalized spectral heart rate variability indices in sleep research: a critical review. *Sleep* 30 913–919. 10.1093/sleep/30.7.913 17682663 PMC1978375

[B25] Calabrò R. S. Naro A. Manuli A. Leo A. Luca R. D. Buono V. L. (2017). Pain perception in patients with chronic disorders of consciousness: What can limbic system tell us? *Clin. Neurophysiol.* 128 454–462. 10.1016/j.clinph.2016.12.011 28160751

[B26] Candelieri A. Cortese M. D. Dolce G. Riganello F. Sannita W. G. (2011). Visual pursuit: within-day variability in the severe disorder of consciousness. *J. Neurotrauma* 28 2013–2017. 10.1089/neu.2011.1885 21770758

[B27] Carney R. M. Blumenthal J. A. Freedland K. E. Stein P. K. Howells W. B. Berkman L. F. (2005). Low heart rate variability and the effect of depression on post–myocardial infarction mortality. *Arch. Intern. Med.* 165 1486–1491. 10.1001/archinte.165.13.1486 16009863

[B28] Celesia G. G. (2013). Conscious awareness in patients in vegetative states: myth or reality? *Curr. Neurol. Neurosci. Rep.* 13:395. 10.1007/s11910-013-0395-7 24048705

[B29] Celesia G. G. Sannita W. G. (2013). Can patients in vegetative state experience pain and have conscious awareness? *Neurology* 80 328–329. 10.1212/WNL.0b013e31827f0928 23255826

[B30] Cerutti S. (1995). *Spectral Analysis of the Heart Rate Variability Signal.* Available at: https://ci.nii.ac.jp/naid/10014992161/ (accessed November 25, 2018). 10.1007/BF02442668 2377001

[B31] Chen Z. Venkat P. Seyfried D. Chopp M. Yan T. Chen J. (2017). Brain–Heart Interaction. *Circ. Res.* 121 451–468. 10.1161/CIRCRESAHA.117.311170 28775014 PMC5553569

[B32] Chennu S. Annen J. Wannez S. Thibaut A. Chatelle C. Cassol H. (2017). Brain networks predict metabolism, diagnosis and prognosis at the bedside in disorders of consciousness. *Brain* 140 2120–2132. 10.1093/brain/awx163 28666351

[B33] Claydon V. E. Krassioukov A. V. (2008). Clinical correlates of frequency analyses of cardiovascular control after spinal cord injury. *Am. J. Physiol. Heart Circ. Physiol.* 294 H668–H678. 10.1152/ajpheart.00869.2007 18024546

[B34] Cobos M. I. Guerra P. M. Vila J. Chica A. B. (2019). Heart-rate modulations reveal attention and consciousness interactions: COBOS ET AL. *Psychophysiology* 56 e13295. 10.1111/psyp.13295 30362275

[B35] Critchley H. D. (2009). Psychophysiology of neural, cognitive and affective integration: fMRI and autonomic indicants. *Int. J. Psychophysiol.* 73 88–94. 10.1016/j.ijpsycho.2009.01.012 19414044 PMC2722714

[B36] Crone J. S. Bio B. J. Vespa P. M. Lutkenhoff E. S. Monti M. M. (2017). Restoration of thalamo-cortical connectivity after brain injury: recovery of consciousness, complex behavior, or passage of time? *J. Neurosci. Res.* 96 671–687. 10.1002/jnr.24115 28801920

[B37] de Lartigue G. (2014). Putative roles of neuropeptides in vagal afferent signaling. *Physiol. Behav.* 0 155–169. 10.1016/j.physbeh.2014.03.011 24650553 PMC4167981

[B38] de Morree H. M. Szabó B. M. Rutten G.-J. Kop W. J. (2013). Central nervous system involvement in the autonomic responses to psychological distress. *Neth. Heart J.* 21 64–69. 10.1007/s12471-012-0351-1 23184602 PMC3547431

[B39] deBoer R. W. Karemaker J. M. Strackee J. (1987). Hemodynamic fluctuations and baroreflex sensitivity in humans: a beat-to-beat model. *Am. J. Physiol.* 253 H680–H689. 10.1152/ajpheart.1987.253.3.H680 3631301

[B40] DeGiorgio C. M. Miller P. Meymandi S. Chin A. Epps J. Gordon S. (2010). RMSSD, a measure of heart rate variability, is associated with risk factors for sudep: the SUDEP-7 inventory. *Epilepsy Behav.* 19 78–81. 10.1016/j.yebeh.2010.06.011 20667792 PMC2943000

[B41] Demertzi A. Antonopoulos G. Heine L. Voss H. U. Crone J. S. de Los Angeles C. (2015). Intrinsic functional connectivity differentiates minimally conscious from unresponsive patients. *Brain* 138 2619–2631. 10.1093/brain/awv169 26117367

[B42] Di Perri C. Bahri M. A. Amico E. Thibaut A. Heine L. Antonopoulos G. (2016). Neural correlates of consciousness in patients who have emerged from a minimally conscious state: a cross-sectional multimodal imaging study. *Lancet Neurol.* 15 830–842. 10.1016/S1474-4422(16)00111-3 27131917

[B43] Di Perri C. Bastianello S. Bartsch A. J. Pistarini C. Maggioni G. Magrassi L. (2013). Limbic hyperconnectivity in the vegetative state. *Neurology* 81 1417–1424. 10.1212/WNL.0b013e3182a43b78 24049132

[B44] Doehner W. Ural D. Haeusler K. G. Čelutkienė J. Bestetti R. Cavusoglu Y. (2018). Heart and brain interaction in patients with heart failure: overview and proposal for a taxonomy. A position paper from the Study Group on Heart and Brain Interaction of the Heart Failure Association. *Eur. J. Heart Fail.* 20 199–215. 10.1002/ejhf.1100 29280256

[B45] Duclos C. Dumont M. Arbour C. Paquet J. Blais H. Menon D. K. (2017). Parallel recovery of consciousness and sleep in acute traumatic brain injury. *Neurology* 88 268–275. 10.1212/WNL.0000000000003508 28003503 PMC5272791

[B46] Ernst G. (2017). Heart-rate variability—more than heart beats? *Front. Public Health* 5:240 10.3389/fpubh.2017.00240 PMC5600971 28955705

[B47] Esler M. (1993). Clinical application of noradrenaline spillover methodology: delineation of regional human sympathetic nervous responses. *Pharmacol. Toxicol.* 73 243–253. 10.1111/j.1600-0773.1993.tb00579.x 8115306

[B48] Faes L. Nollo G. Jurysta F. Marinazzo D. (2014). Information dynamics of brain–heart physiological networks during sleep. *New J. Phys.* 16:105005 10.1088/1367-2630/16/10/105005

[B49] Friedman B. H. (2007). An autonomic flexibility–neurovisceral integration model of anxiety and cardiac vagal tone. *Biol. Psychol.* 74 185–199. 10.1016/j.biopsycho.2005.08.009 17069959

[B50] Gao L. Smielewski P. Czosnyka M. Ercole A. (2016). Cerebrovascular signal complexity six hours after intensive care unit admission correlates with outcome after severe traumatic brain injury. *J. Neurotrauma* 33 2011–2018. 10.1089/neu.2015.4228 26916703

[B51] Garan H. (2009). Heart rate variability in acute myocardial infarction. *Cardiology* 114 273–274. 10.1159/000235567 19690409

[B52] Garbarino S. Lanteri P. Feeling N. R. Jarczok M. N. Quintana D. S. Koenig J. (2019). Circadian rhythms, sleep, and the autonomic nervous system: a position paper. *J. Psychophysiol.* 10.1027/0269-8803/a000236

[B53] Garbarino S. Nobili L. Costa G. (eds) (2014). *Sleepiness and Human Impact Assessment*. Basel: Springer.

[B54] Garbarino S. Sannita W. G. (2015). DoC: a pathophysiological continuum with high variabiity? *Neurology.* 10.13140/RG.2.1.1541.0006

[B55] Giacino J. T. Kalmar K. Whyte J. (2004). The JFK coma recovery scale-revised: measurement characteristics and diagnostic utility. *Arch. Phys. Med. Rehabil.* 85 2020–2029. 10.1016/j.apmr.2004.02.033 15605342

[B56] Goldstein B. DeKing D. DeLong D. J. Kempski M. H. Cox C. Kelly M. M. (1993). Autonomic cardiovascular state after severe brain injury and brain death in children. *Crit. Care Med.* 21 228–233. 8428474 10.1097/00003246-199302000-00014

[B57] Goldstein B. Fiser D. H. Kelly M. M. Mickelsen D. Ruttimann U. Pollack M. M. (1998). Decomplexification in critical illness and injury: relationship between heart rate variability, severity of illness, and outcome. *Crit. Care Med.* 26 352–357. 10.1097/00003246-199802000-00040 9468175

[B58] Grimm T. Kreutz G. (2018). Music interventions in disorders of consciousness (DOC) – a systematic review. *Brain Inj.* 32 704–714. 10.1080/02699052.2018.1451657 29565697

[B59] Gutiérrez J. Machado C. Estévez M. Olivares A. Hernández H. Perez J. (2010). Heart rate variability changes induced by auditory stimulation in persistent vegetative state. *Int. J. Disabil. Hum. Dev.* 9 357–362. 10.1515/IJDHD.2010.041 20566300

[B60] Hagemann D. Waldstein S. R. Thayer J. F. (2003). Central and autonomic nervous system integration in emotion. *Brain Cogn.* 52 79–87. 10.1016/S0278-2626(03)00011-3 12812807

[B61] Hendén P. L. Söndergaard S. Rydenhag B. Reinsfelt B. Ricksten S.-E. Aneman A. (2014). Can baroreflex sensitivity and heart rate variability predict late neurological outcome in patients with traumatic brain injury? *J. Neurosurg. Anesthesiol.* 26 50–59. 10.1097/ANA.0b013e3182a47b62 24064714

[B62] Keren O. Yupatov S. Radai M. M. Elad-Yarum R. Faraggi D. Abboud S. (2005). Heart rate variability (HRV) of patients with traumatic brain injury (TBI) during the post-insult sub-acute period. *Brain Inj.* 19 605–611. 10.1080/02699050400024946 16175814

[B63] Kim S. W. Jeon H. R. Kim J. Y. Kim Y. (2017). Heart rate variability among children with acquired brain injury. *Ann. Rehabil. Med.* 41 951–960. 10.5535/arm.2017.41.6.951 29354571 PMC5773438

[B64] King D. R. Ogilvie M. P. Pereira B. M. Chang Y. Manning R. J. Conner J. A. (2009). Heart rate variability as a triage tool in patients with trauma during prehospital helicopter transport. *J. Trauma* 67 436–440. 10.1097/TA.0b013e3181ad67de 19741382

[B65] Kiryachkov Y. Shelkunova I. Shelkunova I. G. Kolesov D. L. Danilec V. V. (2017). MON-P025: association between heart rate variability measures and energy homeostasis in patients with vegetative status: a prospective clinical cohort pilot study. *Clin. Nutr.* 36:S188.

[B66] Lane R. Mcrae K. Reiman E. Chen K. Ahern G. Thayer J. (2009). Neural correlates of heart rate variability during emotion. *Neuroimage* 44 213–222. 10.1016/j.neuroimage.2008.07.056 18778779

[B67] Laureys S. Celesia G. G. Cohadon F. Lavrijsen J. León-Carrión J. Sannita W. G. (2010). Unresponsive wakefulness syndrome: a new name for the vegetative state or apallic syndrome. *BMC Med.* 8:68. 10.1186/1741-7015-8-68 21040571 PMC2987895

[B68] Laureys S. Faymonville M.-E. Luxen A. Lamy M. Franck G. Maquet P. (2000). Restoration of thalamocortical connectivity after recovery from persistent vegetative state. *Lancet* 355 1790–1791. 10.1016/s0140-6736(00)02271-6 10832834

[B69] Laureys S. Faymonville M. E. Peigneux P. Damas P. Lambermont B. Del Fiore G. (2002). Cortical processing of noxious somatosensory stimuli in the persistent vegetative state. *Neuroimage* 17 732–741. 10.1006/nimg.2002.1236 12377148

[B70] Laureys S. Perrin F. Brédart S. (2007). Self-consciousness in non-communicative patients. *Conscious. Cogn.* 16 722–741. 10.1016/j.concog.2007.04.004 17544299

[B71] Lee Y.-C. Lei C.-Y. Shih Y.-S. Zhang W.-C. Wang H.-M. Tseng C.-L. (2011). “HRV response of vegetative state patient with music therapy,” in *Proceedings of the Annual International Conference of the IEEE Engineering in Medicine and Biology Society*, Boston, MA, 1701–1704. 10.1109/IEMBS.2011.6090488 22254653

[B72] Lehrer P. Eddie D. (2013). Dynamic processes in regulation and some implications for biofeedback and biobehavioral interventions. *Appl. Psychophysiol. Biofeedback* 38 143–155. 10.1007/s10484-013-9217-6 23572244 PMC3699855

[B73] Lehrer P. M. (2007). Biofeedback training to increase heart rate variability. *Princ. Pract. Stress Manag.* 3 227–248.

[B74] Machado C. Korein J. Aubert E. Bosch J. Alvarez M. A. Rodríguez R. (2007). Recognizing a Mother’s voice in the persistent vegetative state. *Clin. EEG Neurosci.* 38 124–126. 10.1177/155005940703800306 17844939

[B75] Majerus S. Bruno M.-A. Schnakers C. Giacino J. T. Laureys S. (2009). “The problem of aphasia in the assessment of consciousness in brain-damaged patients,” in *Progress in Brain Research*, ed. Laureys S. Schiff N. D. Owen A. M. (Amsterdam: Elsevier), 49–61. 10.1016/s0079-6123(09)17705-1 19818894

[B76] Malliani A. (1995). Association of heart rate variability components with physiological regulatory mechanisms. *Heart Rate Var.* 8 202–242.

[B77] Marino S. Bonanno L. Ciurleo R. Baglieri A. Morabito R. Guerrera S. (2017). Functional evaluation of awareness in vegetative and minimally conscious state. *Open Neuroimaging J.* 11 17–25. 10.2174/1874440001711010017 28553427 PMC5427708

[B78] Montano N. Ruscone T. G. Porta A. Lombardi F. Pagani M. Malliani A. (1994). Power spectrum analysis of heart rate variability to assess the changes in sympathovagal balance during graded orthostatic tilt. *Circulation* 90 1826–1831. 10.1161/01.cir.90.4.1826 7923668

[B79] Monti M. M. (2012). Cognition in the vegetative state. *Annu. Rev. Clin. Psychol* 8 431–454. 10.1146/annurev-clinpsy-032511-143050 22224835

[B80] Monti M. M. Rosenberg M. Finoia P. Kamau E. Pickard J. D. Owen A. M. (2014). Thalamo-frontal connectivity mediates top-down cognitive functions in disorders of consciousness. *Neurology* 84 167–173. 10.1212/WNL.0000000000001123 25480912 PMC4336082

[B81] Monti M. M. Sannita W. G. (eds) (2016). *Brain Function and Responsiveness in Disorders of Consciousness.* Berlin: Springer.

[B82] Morris J. A. Norris P. R. Ozdas A. Waitman L. R. Harrell F. E. Williams A. E. (2006). Reduced heart rate variability: an indicator of cardiac uncoupling and diminished physiologic reserve in 1,425 trauma patients. *J. Trauma* 60 1165–1173. 10.1097/01.ta.0000220384.04978.3b 16766957

[B83] Mowery N. T. Norris P. R. Riordan W. Jenkins J. M. Williams A. E. Morris J. A. (2008). Cardiac uncoupling and heart rate variability are associated with intracranial hypertension and mortality: a study of 145 trauma patients with continuous monitoring. *J. Trauma* 65 621–627. 10.1097/TA.0b013e3181837980 18784576

[B84] Nait-Ali A. (2009). *Advanced Biosignal Processing.* Berlin: Springer.

[B85] Napadow V. Dhond R. Conti G. Makris N. Brown E. N. Barbieri R. (2008). Brain correlates of autonomic modulation: combining heart rate variability with fMRI. *Neuroimage* 42 169–177. 10.1016/j.neuroimage.2008.04.238 18524629 PMC2603289

[B86] Naro A. Leo A. Bramanti P. Calabrò R. S. (2015). Moving toward conscious pain processing detection in chronic disorders of consciousness: anterior cingulate cortex neuromodulation. *J. Pain* 16 1022–1031. 10.1016/j.jpain.2015.06.014 26208761

[B87] Norris P. R. Anderson S. M. Jenkins J. M. Williams A. E. Morris J. A. (2008a). Heart rate multiscale entropy at three hours predicts hospital mortality in 3,154 trauma patients. *Shock* 30 17–22. 10.1097/SHK.0b013e318164e4d0 18323736

[B88] Norris P. R. Stein P. K. Morris J. A. (2008b). Reduced heart rate multiscale entropy predicts death in critical illness: a study of physiologic complexity in 285 trauma patients. *J. Crit. Care* 23 399–405. 10.1016/j.jcrc.2007.08.001 18725047

[B89] Norris P. R. Ozdas A. Cao H. Williams A. E. Harrell F. E. Jenkins J. M. (2006). Cardiac uncoupling and heart rate variability stratify ICU patients by mortality: a study of 2088 trauma patients. *Ann. Surg.* 243 804–812. 16772784 10.1097/01.sla.0000219642.92637.fd PMC1570581

[B90] O’Kelly J. Magee W. L. (2013). Music therapy with disorders of consciousness and neuroscience: the need for dialogue. *Nord. J. Music Ther.* 22 93–106. 10.1080/08098131.2012.709269

[B91] Owen A. M. (2014). Disorders of consciousness: diagnostic accuracy of brain imaging in the vegetative state. *Nat. Rev. Neurol.* 10 370–371. 10.1038/nrneurol.2014.102 24934139

[B92] Owen A. M. Coleman M. R. Boly M. Davis M. H. Laureys S. Pickard J. D. (2006). Detecting awareness in the vegetative state. *Science* 313 1402–1402. 10.1126/science.1130197 16959998

[B93] Pagani M. Lombardi F. Guzzetti S. Sandrone G. Rimoldi O. Malfatto G. (1984). Power spectral density of heart rate variability as an index of sympatho-vagal interaction in normal and hypertensive subjects. *J. Hypertens. Suppl.* 2 S383–S385. 6599685

[B94] Papaioannou V. Giannakou M. Maglaveras N. Sofianos E. Giala M. (2008). Investigation of heart rate and blood pressure variability, baroreflex sensitivity, and approximate entropy in acute brain injury patients. *J. Crit. Care* 23 380–386. 10.1016/j.jcrc.2007.04.006 18725044

[B95] Papaioannou V. E. Maglaveras N. Houvarda I. Antoniadou E. Vretzakis G. (2006). Investigation of altered heart rate variability, nonlinear properties of heart rate signals, and organ dysfunction longitudinally over time in intensive care unit patients. *J. Crit. Care* 21 95–103. 10.1016/j.jcrc.2005.12.007 16616632

[B96] Pistoia F. Sacco S. Stewart J. Sarà M. Carolei A. Pistoia F. (2016). Disorders of consciousness: painless or painful conditions?—Evidence from neuroimaging studies. *Brain Sci.* 6:E47 10.3390/brainsci6040047 PMC5187561 27740600

[B97] Rajendra Acharya U. Paul Joseph K. Kannathal N. Lim C. M. Suri J. S. (2006). Heart rate variability: a review. *Med. Biol. Eng. Comput.* 44 1031–1051. 10.1007/s11517-006-0119-0 17111118

[B98] Rapenne T. Moreau D. Lenfant F. Vernet M. Boggio V. Cottin Y. (2001). Could heart rate variability predict outcome in patients with severe head injury? A pilot study. *J. Neurosurg. Anesthesiol.* 13 260–268. 10.1097/00008506-200107000-00016 11426105

[B99] Riganello F. (2016). “Responsiveness and the autonomic control–CNS two-way interaction in disorders of consciousness,” in *Brain Function and Responsiveness in Disorders of Consciousness*, eds Monti M. M. Sannita W. G. (Cham: Springer), 145–155. 10.1007/978-3-319-21425-2_11

[B100] Riganello F. Candelieri A. Quintieri M. Conforti D. Dolce G. (2010). Heart rate variability: an index of brain processing in vegetative state? An artificial intelligence, data mining study. *Clin. Neurophysiol.* 121 2024–2034. 10.1016/j.clinph.2010.05.010 20566300

[B101] Riganello F. Chatelle C. Schnakers C. Laureys S. (2018a). Heart Rate Variability as an indicator of nociceptive pain in disorders of consciousness? *J. Pain Symptom Manage.* 57 47–56. 10.1016/j.jpainsymman.2018.09.016 30267843

[B102] Riganello F. Cortese M. D. Arcuri F. Dolce G. Lucca L. F. Sannita W. G. (2015a). Autonomic nervous system functional state, neuro-rehabiliation, and outcome in disorders of consciousness. *J. Neurotrauma* 32 1071–1077. 25604680 10.1089/neu.2014.3539

[B103] Riganello F. Cortese M. D. Arcuri F. Quintieri M. Dolce G. (2015b). How Can music influence the autonomic nervous system response in patients with severe disorder of consciousness? *Front. Neurosci.* 9:461. 10.3389/fnins.2015.00461 26696818 PMC4674557

[B104] Riganello F. Cortese M. D. Dolce G. Lucca L. F. Sannita W. G. (2015c). The autonomic system functional state predicts responsiveness in DOC. *J. Neurotrauma* 32 1071–1077. 10.1089/neu.2014.3539 25604680

[B105] Riganello F. Cortese M. D. Dolce G. Sannita W. G. (2013). Visual pursuit response in the severe disorder of consciousness: modulation by the central autonomic system and a predictive model. *BMC Neurol.* 13:164. 10.1186/1471-2377-13-164 24195685 PMC3832247

[B106] Riganello F. Dolce G. Sannita W. (2012a). Heart rate variability and the central autonomic network in the severe disorder of consciousness. *J. Rehabil. Med.* 44 495–501. 10.2340/16501977-0975 22660999

[B107] Riganello F. Garbarino S. Sannita W. G. (2012b). Heart rate variability, homeostasis, and brain function: a tutorial and review of application. *J. Psychophysiol.* 26 178–203. 10.1027/0269-8803/a000080

[B108] Riganello F. Garbarino S. Sannita W. G. (2014). Heart rate variability and the two-way interaction between CNS and the central autonomic network. *J. Exp. Clin. Cardiol.* 20 5584–5595.

[B109] Riganello F. Larroque S. K. Bahri M. A. Heine L. Martial C. Carrière M. (2018b). A heartbeat away from consciousness: heart rate variability entropy can discriminate disorders of consciousness and is correlated with resting-state fMRI brain connectivity of the central autonomic network. *Front. Neurol* 9:769. 10.3389/fneur.2018.00769 30258400 PMC6145008

[B110] Riganello F. Macri S. Alleva E. Petrini C. Soddu A. Leòn-Carriòn J. (2016). Pain perception in unresponsive wakefulness syndrome may challenge the interruption of artificial nutrition and hydration: neuroethics in action. *Front. Neurol.* 7:202 10.3389/fneur.2016.00202 PMC5110539 27899911

[B111] Ruiz Vargas E. Sörös P. Shoemaker J. K. Hachinski V. (2016). Human cerebral circuitry related to cardiac control: a neuroimaging meta-analysis: cardiac control. *Ann. Neurol.* 79 709–716. 10.1002/ana.24642 30240034

[B112] Ryan M. L. Thorson C. M. Otero C. A. Vu T. Proctor K. G. (2011). Clinical applications of heart rate variability in the triage and assessment of traumatically injured patients. *Anesthesiol. Res. Pract.* 2011:e416590. 10.1155/2011/416590 21350685 PMC3038414

[B113] Sannita W. G. (2006). Individual variability, end-point effects and possible biases in electrophysiological research. *Clin. Neurophysiol.* 117 2569–2583. 10.1016/j.clinph.2006.04.026 16949340

[B114] Sannita W. G. (2014). Human brain physiology investigated in the disorder of consciousness. *Front. Neurol.* 5:211. 10.3389/fneur.2014.00211 PMC4201104 25368600

[B115] Sannita W. G. (2015). Responsiveness in DoC and individual variability. *Front. Hum. Neurosci.* 9:270 10.3389/fnhum.2015.00270 PMC4432661 26029087

[B116] Saper C. B. (2002). The central autonomic nervous system: conscious visceral perception and autonomic pattern generation. *Annu. Rev. Neurosci.* 25 433–469. 10.1146/annurev.neuro.25.032502.111311 12052916

[B117] Sarà M. Sebastiano F. Sacco S. Pistoia F. Onorati P. Albertini G. (2008). Heart rate non linear dynamics in patients with persistent vegetative state: a preliminary report. *Brain Inj.* 22 33–37. 10.1080/02699050701810670 18183507

[B118] Shaffer F. McCraty R. Zerr C. L. (2014). A healthy heart is not a metronome: an integrative review of the heart’s anatomy and heart rate variability. *Front Psychol.* 5:1040. 10.3389/fpsyg.2014.01040 25324790 PMC4179748

[B119] Shen D. Cui L. Shen Z. Garbarino S. Sannita W. G. Stevens R. D. (2016). Resting brain activity in disorders of consciousness: a systematic review and meta-analysis. *Neurology* 84 1272–1280. 10.1212/WNL.0000000000002274 26755616

[B120] Shi H. Yang L. Zhao L. Su Z. Mao X. Zhang L. (2017). Differences of heart rate variability between happiness and sadness emotion states: a pilot study. *J. Med. Biol. Eng.* 37 527–539. 10.1007/s40846-017-0238-0

[B121] Soddu A. Bassetti C. L. (2017). A good sleep for a fresh mind in patients with acute traumatic brain injury. *Neurology* 88 226–227. 10.1212/WNL.0000000000003529 28003498

[B122] Soddu A. Gómez F. Heine L. Di Perri C. Bahri M. A. Voss H. U. (2015). Correlation between resting state fMRI total neuronal activity and PET metabolism in healthy controls and patients with disorders of consciousness. *Brain Behav.* 6:e00424. 10.1002/brb3.424 27110443 PMC4834945

[B123] Task Force of the European Society of Cardiology and the North American Society of Pacing and Electrophysiology (1996). Heart rate variability: standards of measurement, physiological interpretation and clinical use. *Circulation* 93 1043–1065. 10.1161/01.cir.93.5.1043 8598068

[B124] Thayer J. F. Åhs F. Fredrikson M. Sollers J. J. Wager T. D. (2012). A meta-analysis of heart rate variability and neuroimaging studies: implications for heart rate variability as a marker of stress and health. *Neurosci. Biobehav. Rev.* 36 747–756. 10.1016/j.neubiorev.2011.11.009 22178086

[B125] Thayer J. F. Lane R. D. (2000). A model of neurovisceral integration in emotion regulation and dysregulation. *J. Affect. Disord.* 61 201–216. 10.1016/s0165-0327(00)00338-4 11163422

[B126] Thayer J. F. Lane R. D. (2009). Claude Bernard and the heart–brain connection: further elaboration of a model of neurovisceral integration. *Neurosci. Biobehav. Rev.* 33 81–88. 10.1016/j.neubiorev.2008.08.004 18771686

[B127] Thayer J. F. Sternberg E. (2006). Beyond heart rate variability. *Ann. N. Y. Acad. Sci.* 1088 361–372. 10.1196/annals.1366.014 17192580

[B128] Thome J. Densmore M. Frewen P. A. McKinnon M. C. Théberge J. Nicholson A. A. (2017). Desynchronization of autonomic response and central autonomic network connectivity in posttraumatic stress disorder: CAN Connectivity and HRV in PTSD. *Hum. Brain Mapp.* 38 27–40. 10.1002/hbm.23340 27647521 PMC6866719

[B129] Tobaldini E. Toschi-Dias E. Trimarchi P. D. Brena N. Comanducci A. Casarotto S. (2018). Cardiac autonomic responses to nociceptive stimuli in patients with chronic disorders of consciousness. *Clin. Neurophysiol.* 129 1083–1089. 10.1016/j.clinph.2018.01.068 29486984

[B130] Tonhajzerova I. Ondrejka I. Turianikova Z. Javorka K. Calkovska A. Javorka M. (2012). “Heart rate variability: an index of the brain–heart interaction,” in *Tachycardia*, ed. Yamad T. (Rijeka: Intech), 185–202.

[B131] Valenza G. Duggento A. Passamonti L. Diciotti S. Tessa C. Barbieri R. (2017). “Resting-state brain correlates of instantaneous autonomic outflow,” in *Proceedings of the 39th Annual International Conference of the IEEE Engineering in Medicine and Biology Society (EMBC)*, (Seogwipo: IEEE), 3325–3328. 10.1109/EMBC.2017.8037568 29060609

[B132] Vaschillo E. G. Vaschillo B. Pandina R. J. Bates M. E. (2011). Resonances in the cardiovascular system caused by rhythmical muscle tension: rhythmical muscle tension and resonance. *Psychophysiology* 48 927–936. 10.1111/j.1469-8986.2010.01156.x 21143610 PMC3094735

[B133] Wallin B. G. Charkoudian N. (2007). Sympathetic neural control of integrated cardiovascular function: insights from measurement of human sympathetic nerve activity. *Muscle Nerve* 36 595–614. 10.1002/mus.20831 17623856

[B134] Weir J. Steyerberg E. W. Butcher I. Lu J. Lingsma H. F. McHugh G. S. (2012). Does the extended glasgow outcome scale add value to the conventional Glasgow outcome scale? *J. Neurotrauma* 29 53–58. 10.1089/neu.2011.2137 22026476 PMC3253309

[B135] Wijnen V. J. Heutink M. van Boxtel G. J. Eilander H. J. de Gelder B. (2006). Autonomic reactivity to sensory stimulation is related to consciousness level after severe traumatic brain injury. *Clin. Neurophysiol.* 117 1794–1807. 10.1016/j.clinph.2006.03.006 16793340

[B136] Winchell R. J. Hoyt D. B. (1997). Analysis of heart-rate variability: a noninvasive predictor of death and poor outcome in patients with severe head injury. *J. Trauma Acute Care Surg.* 43 927–933. 10.1097/00005373-199712000-00010 9420107

[B137] Yentes J. M. Hunt N. Schmid K. K. Kaipust J. P. McGrath D. Stergiou N. (2013). The appropriate use of approximate entropy and sample entropy with short data sets. *Ann. Biomed. Eng.* 41 349–365. 10.1007/s10439-012-0668-3 23064819 PMC6549512

